# Breastfeeding Knowledge and Attitudes among Midwifery Diploma Students in Jordan: A Descriptive Study

**DOI:** 10.30476/ijcbnm.2021.88755.1542

**Published:** 2021-10

**Authors:** Diala Altwalbeh

**Affiliations:** Department of Allied Medical Sciences, Faculty of Karak College, Al-Balqa Applied University, Salt, Jordan

**Keywords:** Attitudes, Breastfeeding, Knowledge, Midwifery

## Abstract

**Background:**

Healthcare providers widely support breastfeeding as the optimal form of nutrition for infants. Midwives play a vital role in assisting the mothers to initiate and continue breastfeeding. Therefore, they must acquire proper knowledge accompanied by positive attitudes toward breastfeeding during college education. The main aim of the present study was to assess the knowledge and attitudes of diploma midwifery students toward breastfeeding.

**Methods:**

This is a descriptive cross-sectional study. A short version of the Australian Breastfeeding Knowledge and Attitude Questionnaire (ABKAQ-SF) was distributed among 72 diploma midwifery students from one community college in July 2018, using convenience sampling. Data were analyzed using SPSS software, and the statistical significance was set at P<0.05 level.

**Results:**

Results revealed neutral attitudes (a mean score of 3.02±0.36 out of 5), coupled with a lack of breastfeeding knowledge (mean score was 10.07±2.38 out of 22), particularly knowledge regarding breastfeeding problem management. Midwifery students’ knowledge and attitudes scores were significantly correlated with one another (r=0.68, P=0.001). Demographic variables such as age, year of study, residency place, or personal breastfeeding experiences affected neither the students’ breastfeeding knowledge nor their attitudes.

**Conclusion:**

The findings of this study highlight the need for reforming the curricula of midwifery colleges. Midwifery curricula should provide in-depth knowledge of human lactation physiology and management and give students the basic skills they need to assist breastfeeding women. At the same time, it should focus on the development of supportive and positive attitudes toward breastfeeding.

## INTRODUCTION

When it comes to the best possible nutrition for babies in the earliest stages of their lives, the prevailing opinion is that ‘breast is best’. ^1^ In fact, global organizations, including the World Health Organization (WHO) and the United Nations Children’s Fund (UNICEF) endorse starting breastfeeding within 1 hour of birth. It is also recommended that breastfeeding should continue exclusively in the first six months of life. ^2^ Exclusive breastfeeding is proven to have uncountable health benefits not only for infants but for mothers as well. ^3^

Despite the widespread recommendations and benefits of breastfeeding, there is a global deficit in its practice. Research shows that only 37% of children aged 6 months or less are exclusively breastfed in low and middle-income countries. ^3^ This aversion to breastfeeding might be caused by several physiological, psychological, and social factors. ^4 , 5^

It is quite common in Jordan to breastfeed a child, as shown by the fact that 92% of the children here were breastfed at some stage in their life. Nevertheless, there have been some alarming indicators as well. The rate of exclusive breastfeeding in infants aged six months is less than optimal and is falling steadily. Several Jordanian population and family health surveys showed that exclusive breastfeeding decreased from 40% in 2007 to 26% in 2018. ^6^ When surveyed, Jordanian mothers provided several reasons for their inability to breastfeed exclusively. These reasons include insufficient milk, C-sections, return to work, and a preference for bottle feeding as a routine practice. They also cited breast problems, tiredness and shorter periods between pregnancies as reasons to avoid exclusive breastfeeding. ^7 , 8^ Two previous studies in Jordan showed negative attitudes toward breastfeeding practice. ^9 , 10^ The majority of the participating women did not plan to exclusively breastfeed their babies in the first six months. ^9^ Several studies from Jordan have pinpointed the insufficient counselling and support for mothers during their hospital stay or when they show up for antenatal care; for example, only 66% of mothers are given postnatal counseling on breastfeeding. ^6 , 7^ Some authors have argued that there is ineffectual application of breastfeeding policies and an absence of health promotion policies that aim to improve attitudes towards breastfeeding among women of child-bearing age. ^7 , 10^ At the same time, there is deficit in the training of healthcare providers. ^7 , 11^

An effective way to enhance breastfeeding rates and attitudes is to prepare healthcare providers who know how to promote, protect, and support breastfeeding among the local population. ^12^ A Cochrane Review by Mcfadden concluded that support from healthcare providers could be effective in prolonging the period of breastfeeding. ^13^ Unfortunately, there is evidencethat many healthcare providers suffer from low confidence levels and unsatisfactory clinical competence, attitude, and knowledge of breastfeeding. ^14 - 16^

Midwives are one of the frontline healthcare providers in the struggle to promote exclusive breastfeeding. They can provide the support and information that the mothers need to ensure the health of their babies. ^17^ Recent research shows that women who had a midwife accompanying them as they gave birth had a significantly higher likelihood of breastfeeding exclusively, not only at the time of discharge, but also 3-months postpartum. ^18^ Another study reported that women who are given antenatal care by a midwife are more prone to expressing their willingness to breastfeed as compared to those who get care from an obstetrician. ^19^ Conclusively, the role of a midwife seems essential when it comes to supporting mothers to breastfeed. Therefore, it is crucial to give midwives the tools to succeed in their roles effectively. Preparation must begin in undergraduate schools and colleges. It is suggested that midwifery students should know different methods of breastfeeding and understand the physiological process of lactation. When this type of education is combined with positive reinforcement, non-judgmental attitudes, effective communication and practical clinical skills, midwives are better able to assist the new mothers to provide optimal care for their infants. ^20 , 21^

The knowledge and attitudes of healthcare providers towards breastfeeding have been the subject of research over the years in an effort to improve the outcomes regarding breastfeeding. ^14 , 22^ The results of a systematic review study on the general knowledge and attitudes of healthcare students regarding breastfeeding showed that most of them had focused on nursing and medical students in countries outside of Jordan. It was concluded that healthcare students (including midwifery students) had moderate results in breastfeeding attitudes, and inadequate breastfeeding knowledge, especially when it came to assessment and problems management. ^23^ The authors of the review summarized the factors that influence knowledge and attitudes regarding breastfeeding among the students. These include the timing and content of the maternal and child health courses, past experiences, sex, culture, and legislation. The results of another study on breastfeeding knowledge and attitudes among breastfeeding women, peer supporters, and midwifery students revealed that, for midwifery students, the knowledge and attitude scores were considerably higher than breastfeeding women but lower than peer supporters. ^24^ To the best of our knowledge, there was no study on breastfeeding knowledge and attitudes of midwifery students in Jordan or anywhere else in the Arab world. Therefore, the aim of this study was to fill the gaps in the existing literature. This study was designed to assess the knowledge and attitudes of diploma midwifery students toward breastfeeding. It also evaluated the perceived confidence of students in meeting the needs of breastfeeding women, and their satisfaction with college breastfeeding education.

## METHODS

This is a descriptive cross-sectional study; the participants were diploma midwifery students from Karak community college selected through convenience sampling. Al-Balqa Applied University, through its community colleges, offers a diploma in midwifery for students who have completed 96 credit hours over three years. According to the registration office at Al-Balqa Applied University, in July 2018, there were 465 eligible students for this study in all its related community colleges. The sample size was calculated using an online statistics calculator based on the formula: n=N*X/(X+N–1). Using a margin of error: 5%, confident level: 95%, population size of 465, and a sample proportion of 10%, we estimated the sample size as107 students. ^25^ The accessible population was identified as midwifery students from Karak community college. Eligible participants met the following inclusion criteria: passing the theoretical and clinical courses in neonatology, child health nursing, and basic and applied nutrition courses and signing a consent form. At Karak community college, a group of 140 students was considered eligible for participation. Twenty students underwent a pilot study, so they were excluded from the sample frame, and 20 students refused to participate in the study. From the remaining 100 students, 10 did not return the questionnaire and 18 submitted incomplete questionnaires where more than 20% of data were missing, so they were excluded. Sample mean substitution procedure was applied if the missing data was less than 20%; it involved replacing missing data for a case on a variable with the overall sample mean of that variable. ^26^ The researcher ended with only 72 completed questionnaires.

For the purpose of this study, ethical approval was obtained from the ethics and scientific committee at Karak College (reference number: ESC-3/2018). Additionally, the author had the permission to use of the data collection tool. A list containing the names of all students eligible for the study was received from the registration office of Karak College. These students were then attended theoretical classes and were informed about the purpose of the study. It was clearly ensured that their participation was voluntary and their responses on the questionnaire would remain anonymous. They were required to fill out non-identifying data, which would only be used for research purposes. Furthermore, the students were told that they could withdraw whenever they decide and this would have no consequence. The students who agreed to these terms were asked to sign a consent form, and later they were given research questionnaires to fill out. 

A short version of the Australian Breastfeeding Knowledge and Attitude Questionnaire (ABKAQ-SF) was used for the purpose of this study. It was developed by Brodribb and colleagues. The ABKAQ-SF showed a high reliability for assessing breastfeeding-related knowledge (Cronbach’s alpha was 0.83) and attitudes (Cronbach’s alpha was 0.84). ^27^ Three general practitioners (GPs) and one researcher with adequate education, experience and expertise on breastfeeding were asked to evaluate the content and face validity of the questionnaire used. ^28^ There are 48 items in this adapted questionnaire. Eight items ask about the students’ demographic characteristics, their perceived confidence in their abilities to manage breastfeeding challenges, and their satisfaction regarding breastfeeding content in the academic curriculum. Next, 18 items are about breastfeeding attitudes, and additional 22 items assess the breastfeeding knowledge of the participants. The 22 questions regarding breastfeeding knowledge cover the benefits and physiology of breastfeeding, as well as breastfeeding management. The knowledge section asks questions from the ‘correct-incorrect’ and ‘don’t know’ categories, and 11 of these were false items. Correct answers receive 1 point each, while the incorrect and don’t know answers receive no points. The total knowledge score ranges from 0 to 22, and higher scores indicate a more in-depth knowledge of breastfeeding. The responses to the18 items that explore breastfeeding attitudes were scored according to the 5-point Likert scale, where 1=strongly disagree and 5=strongly agree. Fourteen items in this part were negatively scored. A higher score revealed positive attitudes towards breastfeeding. 

The questionnaire was written and presented to the participants in its original form in the English language, as this is the language of education for all midwifery courses in Jordan. To ensure that the questionnaire is reliable and clear to answer, and to determine the time it takes to complete, it was pilot tested with twenty students who were excluded from the original study. 

The Statistical Package for Social Sciences (SPSS version 18.0.0) was used to analyze the data. The demographic data, knowledge and attitude results were characterized using descriptive statistics, while correlation and t-test were used to distinguish significant relationships and differences. Statistical significance was set at P 0.05 level. Data is shown as a fraction of participants or mean±SD.

## RESULTS

During data collection phase, 100 students received the questionnaires; 72 were filled and returned. All participants were females as midwifery colleges in Jordan only accept female students. Their age ranged between 18 and 26 years with a mean of 19.90±1.66. Nearly 54% of the participants were in the second year, and 46% were in the third year of their studies. 85% were from Karak City and 15% were from Tafiela. Out of all the students surveyed, only 3 (4.17%) had previous personal breastfeeding experience. 

In general, the mean score of breastfeeding knowledge was 10.07±2.38 points out of 22 (range from 6 to15), representing 45.8% of the total score. Table 1 shows that midwifery students in this study had good knowledge about breastfeeding benefits and some basics of lactation physiology; for example, 88.89% knew that women who breastfeed had a lower incidence of premenopausal breast cancer and that breastfed babies had fewer ear infections. A majority (73.61%) knew that high prolactin levels were necessary to initiate lactation. More than three-fourths of the students (79.17%) recommended that mothers should continue breastfeeding for 2 years, and 72.22% knew that solid food should be introduced to breastfed infants at 6 months of age. There was, however, a noticeable lack of knowledge about the management of breastfeeding problems. Less than 10% of students knew that Amoxicillin is not the drug of choice for treating mastitis in a postpartum woman. About 89% of students incorrectly believed that women with cracked nipples should rest the nipples for 24 hours after expressing the milk. Nearly 83% wrongly understood that antenatal nipple preparation could prevent nipple soreness in the first week postpartum. Similarly, 81.94% mistakenly believed that a nipple shield was useful in case the infant could not attach to the breast. Furthermore, only 22.22% understood how to manage an oversupply of breast milk. In addition, some misinformation regarding breastfeeding and weaning was also prevalent; a large percentage of students thought that being pregnant or taking prescribed medications were reasons for weaning the child. The average percent-correct scores for breastfeeding knowledge subscales were computed and compared, as shown in Table 2. The highest percent-correct score was for benefits subscale (0.82±0.08), while the lowest was for breastfeeding management subscale (0.22±0.12). Paired t-test revealed significant differences in the average percent-correct scores between the three subscales. A positive moderate relationship was detected between breastfeeding physiology and management (r=0.43, P<0.001). 

**Table 1:** Number and frequency of correct and incorrect answers for midwifery students’ breastfeeding knowledge (n=72)

Variables	Correct answers N (%)	Incorrect answers N (%)
Breastfeeding management subscale		
1. Breastfed infants require extra water in hot weather. *	25 (34.72)	47 (65.28)
2. A breastfeeding woman should be advised to wean if she becomes pregnant. *	33 (45.83)	39 (54.17)
3. Amoxycillin is the drug of choice to treat mastitis in a woman 3 months postpartum. *	7 (9.72)	65 (90.28)
4. All women with cracked nipples should express their milk and rest the nipples for 24 hrs.*	8 (11.11)	64 (88.89)
5. Antenatal nipple preparation prevents nipple soreness in the first week postpartum. *	12 (16.67)	60 (83.33)
6. A nipple shield should be used if there are any problems with the infant attaching to the breast. *	13 (18.06)	59 (81.94)
7. In most cases a breastfeeding mother must temporarily wean her baby while she is taking prescription medications. *	15 (20.83)	57 (79.17)
8. Only feeding from one breast at each feed is a management option for a woman with an oversupply of breast milk.	16 (22.22)	56 (77.78)
Physiology subscale		
9. It is expected that breastfed infants will regain their birthweight by two weeks of age.	40 (55.56)	32 (44.44)
10. is normal for an adequately breastfed 2-week-old infant to only pass a bowel motion every 3 days or so. *	47 (65.28)	25 (34.72)
11. Breastfeeding is contraindicated for women with Hepatitis C.*	17 (23.61)	55 (76.39)
12. Increasing her fluid intake will increase a mother’s milk supply*.	21 (29.17)	51 (70.83)
13. High maternal prolactin levels are essential for the initiation of lactation.	53 (73.61)	19 (26.39)
14. Introducing complementary feeds (water or formula) interferes with the establishment of breastfeeding.	27 (37.50)	45 (62.5)
15. The nutritional content of breast milk changes throughout a breastfeed.	51 (70.83)	21 (29.17)
16. The most common cause of cracked nipples is poor positioning and attachment of the infant at the breast.	31 (43.06)	41 (56.94)
17. Growth of breastfed infants differs from that of formula fed infants.	48 (66.67)	24 (33.33)
18. A ‘top-up’ bottle after each breastfeed is the best way to manage an infant who is not gaining weight adequately. *	24 (33.33)	48 (66.67)
Benefits subscale		
19. Formula fed infants have more ear infections than breastfed infants.	64 (88.89)	8 (11.11)
20. Women who have breastfed have a lower incidence of premenopausal breast cancer.	64 (88.89)	8 (11.11)
21. Around what age do you recommend solids be introduced to a breastfed infant? True answer: 6 months.	52 (72.22)	20 (27.78)
22. For how long do you recommend to a mother that she continue to breastfeed her infant? True answer: 2 years and beyond.	57 (79.17)	15 (20.83)

* False statement.

**Table 2:** Comparison and correlation between the average percentages of correct scores of midwifery students’ breastfeeding knowledge subscales

Breastfeeding knowledge subscales	Mean±SD	T	P	r	P
Pair 1: Physiology Breastfeeding management	0.50±0.19	13.12	<0.001 *	0.43	<0.001**
	0.22±0.12				
Pair 2: Benefits Breastfeeding management	0.82±0.08	10.23	<0.001 *	0.02	0.85
	0.22±0.12				
Pair 3: Physiology Benefits	0.50±0.19	7.83	<0.001 *	0.03	0.79
	0.82±0.08				

* t test is statically significant if P value is <0.05;

** Pearson’s r coefficient is statically significant if P value is < 0.05.

As shown in Table 3, midwifery students in this study revealed a neutral attitude toward breastfeeding with an overall mean score of 3.02±0.36 out of 5. Interestingly, 94% of the responding students showed disagreement with the statement of “infant formula is as healthy for an infant as breast milk”. Meanwhile, only 11.1% agreed that breastfeeding was more convenient than formula feeding. About 88.9% believed that breastfeeding provided health benefits for infants that could not be provided by infant formula, and 83.3% understood that breastfeeding increased the mother-infant bonding. Only 5.6% of the students disagreed that formula feeding was a good choice for mothers who planned to go out to work. 

**Table 3:** Mean and frequency of midwifery students’ breastfeeding attitudes (n=72)

Variables	Mean±SD	Positive attitudes (N%)
1. Infant formula is more easily digested than breast milk. *	3.17±1.39	40 (55.6)
2. Breast milk is the ideal food for babies. **	3.83±1.02	44 (61.1)
3. Formula feeding is a good way of letting fathers care for the baby. *	2.24±1.14	16 (22.2)
4. Breastfeeding & formula feeding are both equally acceptable methods of feeding infants. *	3.69±1.11	46 (63.9)
5. Breastfeeding increases mother-infant bonding.	4.22±0.98	60 (83.3)
6. Breastfeeding provides health benefits for infants that cannot be provided by infant formula. **	4.44±0.69	64 (88.9)
7. Mothers who smoke should formula feed their babies. *	2.68±1.22	21 (29.2)
8. Breastfeeding is incompatible with working outside the home. *	2.50±1.27	20 (27.8)
9. Fathers feel left out if a mother breastfeeds. *	2.54±1.16	19 (26.4)
10. Breastfed babies need to be fed too often. *	2.42±1.02	16 (22.2)
11. Infant formula is as healthy for an infant as breast milk. *	4.19±0.64	68 (94.4)
12. Breastfeeding is more convenient than formula feeding. **	1.94±1.03	8 (11.1)
13. Formula feeding is the better choice if the mother plans to go out to work. *	2.28±0.88	4 (5.6)
14. The benefits of breast milk last only as long as the baby is breastfed. *	3.33±0.89	36 (50.0)
15. A mother who occasionally drinks alcohol should not breastfeed her baby. *	2.06±0.79	4 (5.6)
16. Formula feeding is more reliable because you can calculate the exact quantity of milk the baby is getting. *	2.44±0.96	12 (16.7)
17. Current infant formulas are nutritionally equivalent to breast milk. *	3.67±0.82	40 (55.6)
18. Women should not breastfeed in public places such as restaurants. *	2.72±1.33	24 (33.3)

* Positive attitudes calculated as the sum of disagree and strongly disagree;

** Positive attitudes calculated as the sum of agree and strongly agree

In general, there was a moderate relationship between the knowledge and attitude scores of the students (*r*=0.68, P=0.001). This study indicated that the demographic variables such as age, year of study, residency place, or personal breastfeeding experiences affected neither the students’ breastfeeding knowledge nor their attitudes, as shown in Tables 4 and 5. Significant positive relationships existed between the students’ perceived confidence in breastfeeding management abilities and breastfeeding knowledge, attitudes, and their satisfaction with breastfeeding education (r=0.50, 0.35, 0.64, P<0.001), respectively. At the same time, a positive relationship was found between satisfaction with breastfeeding education and breastfeeding knowledge and attitudes (r=0.61, 0.38, P<0.001), respectively, as shown in Table 5. 

**Table 4:** Assessment of the mean score of knowledge, attitudes, perceived confidence and satisfaction with breast feeding education according to some sociodemographic characteristics of midwifery students (n=72)

Year of education	Second year	Third year	t	P*
	Mean±SD	Mean±SD		
Breastfeeding knowledge	9.90±2.46	10.28±2.30	-0.66	0.51
Breastfeeding attitudes	3.04±0.38	2.99±0.33	0.57	0.57
Perceived confidence	2.26±0.93	1.91±1.04	1.49	0.14
Satisfaction with breastfeeding education	2.03±0.93	2.03±0.77	-0.02	0.98
**Residency**	**Karak**	**Tafiela**		
Breastfeeding knowledge	10.13±2.46	9.73±1.96	-0.52	0.61
Breastfeeding attitudes	3.04±0.37	2.92±0.30	-0.97	0.34
Perceived confidence	2.15±1.05	1.82±0.60	-1.45	0.16
Satisfaction with breastfeeding education	2.05±0.92	1.91±0.30	-0.50	0.62
**Personal experience with breastfeeding**	**Yes**	**No**		
Breastfeeding knowledge	13.00±1.00	10.81±1.99	1.88	0.07
Breastfeeding attitudes	3.35±0.16	3.12±0.29	1.39	0.17
Perceived confidence	3.00±1.00	2.21±0.98	1.36	0.18
Satisfaction with breastfeeding education	3.00±0.99	2.27±0.74	1.63	0.11

* t test is statically significant if P value is <0.05.

**Table 5:** The Correlation between breastfeeding knowledge, attitudes, perceived confidence, satisfaction with breast feeding education and age

Variables	Breastfeeding knowledge	Breastfeeding attitudes	Perceived confidence	Satisfaction with breastfeeding education	Age
Breastfeeding knowledge	−				
Breastfeeding attitudes	0.68*	−			
Perceived confidence	0.50*	0.35*	−		
Satisfaction with breastfeeding education	0.61*	0.38*	0.64*	−	
Age	0.15	0.08	0.19	0.17	−

* Correlation (Pearson’s r) is significant at the 0.01 level.

On average, the level of confidence was low. The mean score was 2.10±0.10 out of 5. About 88% of the midwifery students reported that they did not receive satisfactory breastfeeding education and training in their midwifery program with a mean of 2.03±0.86 out of 5. 

## DISCUSSION

The results reflected a relatively weak knowledge score (10.07±2.38) with a majority of participants (54.2%) answering more than half of the questions inaccurately. This score is low compared to midwifery students from the United Kingdom, ^24^ nursing students from the USA ^29 - 31^ and Egypt, ^12^ medical and nursing students from Saudi Arabia ^32 , 33^ and India, ^34^ and medical students from Nigeria ^35^ and Jordan. ^36^

Students showed a deficiency of knowledge about management of breast problems such as mastitis, cracked nipples, milk oversupply and appropriate conditions for weaning. The findings of the current study confirm the association between the students’ knowledge regarding breastfeeding physiology and management of its related problems. This finding is consistent with those of earlier studies from Egypt and USA. ^12 , 31^ The lack of breastfeeding knowledge in the current study reflects the shallow and inadequate breastfeeding-related content of the current midwifery curriculum. This assumption was strengthened by AL-Nuaimi and colleagues in a 2019 study from Jordan, in which 60% of participating nurses and midwives showed lack of knowledge in physiology, management, and problems of breastfeeding despite reporting that they received relevant education at their faculties. ^16^ Another reason for the low knowledge score could be the lack of clinical experience in a clinical setting to help enhance their breastfeeding management knowledge and skills. For example, in this study 80% reported that during clinical training, they never saw a mother with breastfeeding-related problems. 

Midwifery students’ attitudes are essential in forming their approach to mothers who present with breastfeeding questions and concerns. The findings of this study reflect neutral attitudes toward breastfeeding. The breastfeeding attitude mean score was 3.02±0.36. This score was lower than other Jordanian undergraduate students ^37 , 38^ and even lower than that of Jordanian mothers. ^11 , 39^ This result was unexpected because Jordanian culture promotes breastfeeding and most Jordanians adhere to Islam as a religion and follow the tenets of the Qur’an, which supports breastfeeding. The Holy Qur’an states that ‘Mothers shall give suck to their children for two whole years, for those who desire to complete the term of sucking’ (Qur’an, 2:233). ^40^ The lowest score belonged to “Breastfeeding is more convenient than formula feeding’, which was expected to be one of the highly scored attitudes. This finding reflects the high acceptance for formula feeding in the Jordanian communities, as seen in the Al-Sagarat study which revealed that most participating Jordanian women actively planned to use bottle formula to feed their babies. ^9^ Socioeconomic factors could be blamed for this issue. Altamimi and Al-Ali have argued that in Jordan, as in other Middle Eastern countries, better education, and employment opportunities for women $\underset{\_\_\_\_\_\_\_\_\_\_\_\_\_\_\_\_\_\_\_\_\_\_\_}{\mathrm{in the last few years}}$ impacted traditional views on infant feeding. ^37 , 39^ It seems that midwifery students in the current study were affected by their local community culture since most of them were from the towns of Karak and Tafelah where breastfeeding rates were the lowest in Jordan. ^6^

This study revealed that knowledge and attitudes are associated with one another. Therefore, it is crucial to concentrate on education and training for midwifery students to develop positive attitudes about breastfeeding. The literature suggests that prevailing attitudes and personal opinions may become obstacles to learning for students. ^41^ Midwifery education institutions in the UK realized this; consequently, students from different social and cultural backgrounds were introduced to activities that would encourage them to be open to unfamiliar ideas regarding breastfeeding. These activities placed the students in situations that challenged their views, including engaging in group discussions and insightful exercises. By the end of their educational program, students with earlier negative attitudes concerning infant breastfeeding exhibited positivity and confidence in their ability to offer scientific information to new parents. ^41^

In contrast to the high level of confidence displayed by nursing students from Egypt, ^12^ the findings of the current study revealed that midwifery students had very little confidence in their abilities to assist the women who present with breastfeeding problems. However, there was a significant positive relationship between the level of confidence and both knowledge and attitude. It seems that students in this study were well aware that they had a knowledge deficit regarding breastfeeding. Similar to a previous study from Jordan, many participants here were dissatisfied with the breastfeeding education offered in nursing and midwifery college. ^16^

The results of this study highlight the need for reforming the curricula of midwifery colleges. Midwifery curricula need to provide in-depth knowledge of human lactation physiology and management, and should give students the basic skills they need to assist breastfeeding women. At the same time, it should focus on the development of supportive and positive attitudes. For more consistent practice, it is advisable to develop national standards for human lactation and breastfeeding courses for all healthcare students in Jordan, as recommended by WHO. ^42^

Although the current study was the first in Jordan and Arab world to assess breastfeeding knowledge and attitudes among midwifery diploma students, it clearly has limitations. The major limitations are the use of convenience sampling and limited number of midwifery students from one public community college, which limits the generalizability of the results. 

## CONCLUSION

This study revealed a lack of knowledge of breastfeeding, coupled with neutral attitudes, low confidence in breastfeeding management and low level of satisfaction with breastfeeding education among diploma midwifery students in Jordan. It should be remembered that the study is based on convenience sampling and small number of students. Its recommended that this study should be repeated with a larger sample of students from several colleges.
